# Protein Tyrosine Phosphatase 1B (PTP1B) Deficiency Substantially Attenuates Glomerular Injury in Endothelial Nitric Oxide Synthase (eNOS)-Deficient Diabetic Mice

**DOI:** 10.7759/cureus.78207

**Published:** 2025-01-29

**Authors:** Daisuke Katagiri, Shinya Nagasaka, Keiko Takahashi, Akira Shimizu, Raymond C Harris, Takamune Takahashi

**Affiliations:** 1 Nephrology, Vanderbilt University Medical Center, Nashville, USA; 2 Analytic Human Pathology, Graduate School of Medicine, Nippon Medical School, Tokyo, JPN

**Keywords:** diabetic nephropathy (dn), endoplasmic reticulum stress (er-stress), endothelial nitric oxide synthase, insulin signaling, podocyte, protein tyrosine phosphatase 1b

## Abstract

Background

Deficiency of endothelial nitric oxide synthase (eNOS) accelerates diabetic nephropathy (DN); however, the underlying mechanisms are incompletely understood. Given that nitric oxide inactivates protein tyrosine phosphatase 1B (PTP1B), a critical negative regulator of insulin signaling, we hypothesized that eNOS deficiency activates PTP1B; this reduces insulin signaling and worsens glomerular injury in DN.

Methods

PTP1B/eNOS double knockout (DKO) mice were generated and compared to eNOS knockout (KO) mice. Diabetes was induced at eight weeks of age by low-dose streptozotocin injections, and phenotypic analyses were performed at 10 and 22 weeks after streptozotocin administration.

Results

Although no differences were found in blood glucose, blood pressure, or left kidney weight-to-body weight ratio between the diabetic DKO and eNOSKO mice, albuminuria was largely reduced in DKO mice. Histological, Immunofluorescence, and immunohistochemical investigations showed substantially milder mesangial expansion and mesangiolysis and higher podocyte numbers and nephrin expression in DKO mice. Spliced X-box binding protein 1 (sXBP-1) expression was greatly increased, and C/EBP-homologous protein (CHOP) was decreased in the podocytes of DKO mice.

Conclusions

PTP1B deficiency substantially reduces glomerular injury in diabetic eNOSKO mice. Enhanced insulin signaling and improved endoplasmic reticulum (ER) stress in podocytes were suggested as a possible mechanism.

## Introduction

Endothelial nitric oxide synthase (eNOS)-derived nitric oxide (NO) is crucial for vascular homeostasis, and its reduction plays a key role in the progression of diabetic nephropathy (DN) [1,2]. The diabetic mice that lack eNOS develop severe glomerular injury that, in part, mirrors human DN [2].

Protein tyrosine phosphatase 1B (PTP1B) is a ubiquitously expressed cytoplasmic PTP that negatively regulates insulin signaling [3]. PTP1B knockout (KO) mice show resistance to obesity and diabetes due to enhanced insulin sensitivity [4]. Given that NO negatively regulates PTP1B activity [5,6], we hypothesized that eNOS deficiency activates PTP1B and reduces insulin signaling in glomerular cells, advancing DN.

To test this hypothesis, we generated diabetic mice that lack both eNOS and PTP1B. Our data demonstrates that PTP1B deficiency substantially attenuates glomerular injury in eNOS-deficient diabetic mice, in part, by restoring endoplasmic reticulum (ER) stress in podocytes. This study demonstrates, for the first time, that PTP1B mediates advanced diabetic glomerular injury induced by eNOS deficiency, implicating PTP1B in molecular targets in DN therapy.

## Materials and methods

Animal model and phenotypic analysis

PTP1BKO (#032240) and eNOSKO (#002684) mice with C57BL/6J strain were purchased from Jackson Laboratory (Bar Harbor, ME). Double knockout (DKO) mice were generated by crossbreeding and compared to eNOSKO mice. Male mice were used for the study. Diabetes was induced at eight weeks of age by injecting streptozotocin (STZ) (50 mg/kg) intraperitoneally for five consecutive days and confirmed (blood glucose > 300 mg/dL) at two weeks after injections. Their phenotypes were assessed at 10 and 22 weeks after STZ injections, as described previously [7]. In brief, blood glucose was measured after a six-hour fast using glucose test strips (Roche Applied Science, Indianapolis, IN). Twenty-four-hour urine was collected from individually caged mice using polycarbonate metabolic cages (Tecniplast, Buguggiatte, Italy), and urinary albumin and creatinine levels were measured using the enzyme-linked immunosorbent assay (ELISA) kits (Exocell Inc., Newtown Square, PA). Systolic blood pressure was measured at least five times in trained conscious mice using a tail-cuff monitor (IITC Life Science, Woodland Hills, CA), and the mean value was calculated in each mouse. Plasma creatinine levels were determined using underivatized stable isotope dilution LC-MS/MS at the UAB O’Brien Center (Birmingham, AL).

Histological, immunofluorescence, and immunohistochemical assessment

Anti-nephrin (AF3159; R&D Systems, Minneapolis, MN), anti-C/EBP-homologous protein (CHOP) (#2895; Cell Signaling Technology, Danvers, MA), anti-Wilms’ tumor gene 1 (WT1) protein (DyLight 550, #857; Novus Biologicals, Littleton, CO), and anti-spliced X-box binding protein 1 (sXBP-1) (MAB4257, R&D Systems) were used for immunostaining. The mice were anesthetized with 1-5% isoflurane in oxygen and kidneys were perfused via the left ventricle with PBS and fixed in 10% neutral buffered formalin overnight. Paraffin sections were stained with periodic acid-Schiff, and mesangial expansion and mesangiolysis were assessed, as described [7,8]. For CHOP immunohistochemistry, a nonspecific reaction for horseradish peroxidase was blocked using 3% hydrogen peroxide in methyl alcohol. Antigen retrieval was carried out by microwave with 0.01M citrate buffer (pH 6.0) for 20 minutes and blocked with 5% goat serum (30 minutes), 5% skim milk (one hour), and Mouse On Mouse kit (one hour; Vector Laboratories, Burlingame, CA) at room temperature (RT). Sections were then incubated with a primary antibody overnight at 4 °C. The immunoreactions were detected using an ABC system (Vector Laboratories, Burlingame, CA) and visualized using chromogen reaction, 3, 3′- diaminobenzidine tetrahydrochloride (Vector Laboratories). CHOP-positive areas were measured using the sections stained without a primary antibody as a background. For sXBP-1 immunostaining, cryostat sections were fixed in 100% acetone for 10 minutes at -20 °C, blocked with 5% skim milk, and incubated with sXBP-1 and WT1 antibodies and fluorescence-labeled secondary antibodies. The glomeruli were photographed using light or fluorescence microscopy, and immunoreactivity was quantified using the ImageJ package (NIH, Bethesda, MD). At least 15 randomly selected cortical glomeruli were examined per mouse. Nephrin-positive areas and WT1-positive cells in glomerular cross-section were assessed by immunofluorescence staining, as described [7].

Statistical analysis 

Data are expressed as mean values ± standard error of the mean (SEM). Statistical analyses were performed using Prism4 software (GraphPad Software Inc., La Jolla, CA). Differences between experimental groups were determined using an unpaired student’s t-test. Statistical significance was set at p < 0.05.

## Results

**Figure 1:**
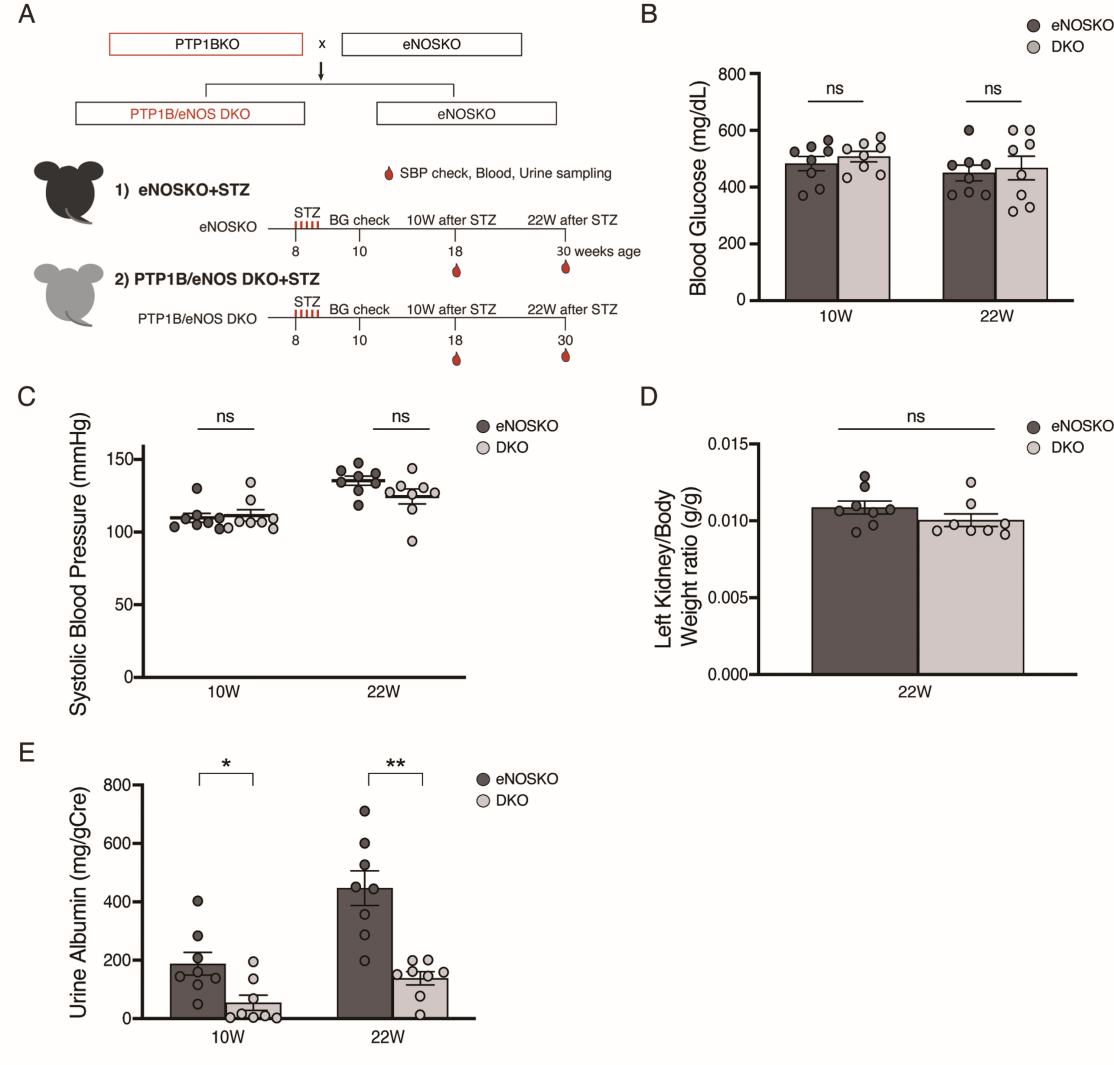
Experimental protocol and phenotypic analyses of diabetic PTP1B/eNOS DKO mice A: Animal model and experimental protocol. B: Fasting blood glucose. C: Systolic blood pressure. D: Left kidney weight-to-body weight ratio. E: Albuminuria. Data are expressed as mean values ± SEM. *p < 0.05, **p < 0.01 vs. diabetic eNOSKO group. N=8 per group. PTP1BKO, PTP1B knockout; eNOSKO, eNOS knockout; DKO, double knockout; STZ, streptozotocin; BG, blood glucose; 10W, 10 weeks after STZ injections; 22W, 22 weeks after STZ injections; SBP, systolic blood pressure

Figure 1A shows the experimental protocol. DKO and eNOSKO mice were generated by crossbreeding, and diabetes was induced at eight weeks of age with low-dose STZ injections. Phenotypic analyses were performed at 10 and 22 weeks after injections. Kidneys were sampled at the end of the study. Since C57BL6/J strain mice do not develop evident DN without eNOS deficiency [9,10], in this study, we assessed the effects of PTP1B deficiency only in eNOSKO mice. As shown in Figures 1B-1D, there were no differences in blood glucose, systolic blood pressure, or left kidney weight-to-body weight ratio between the diabetic DKO and eNOSKO mice. However, albuminuria was significantly reduced in DKO mice at both 10 and 22 weeks (Figure 1E). A significant difference was not observed in plasma creatine level at 22 weeks between the two groups (STZ-eNOSKO vs. STZ-DKO; 0.28 ± 0.13 vs. 0.18 ± 0.10 mg/dL, p=0.99, N=8 per group). Diabetic eNOSKO mice show reduced renal blood flow and limited renal hypertrophy, perhaps due to vasoconstriction and structural vascular defects [8,11,12]. Furthermore, insulin induces vasodilation through eNOS [13]; this action does not happen in the setting of eNOS deficiency. A potential explanation for the lack of differences in plasma creatinine and left kidney weight-to-body weight ratio is that PTP1B deficiency does not improve renal hemodynamics in diabetic eNOSKO mice.

**Figure 2:**
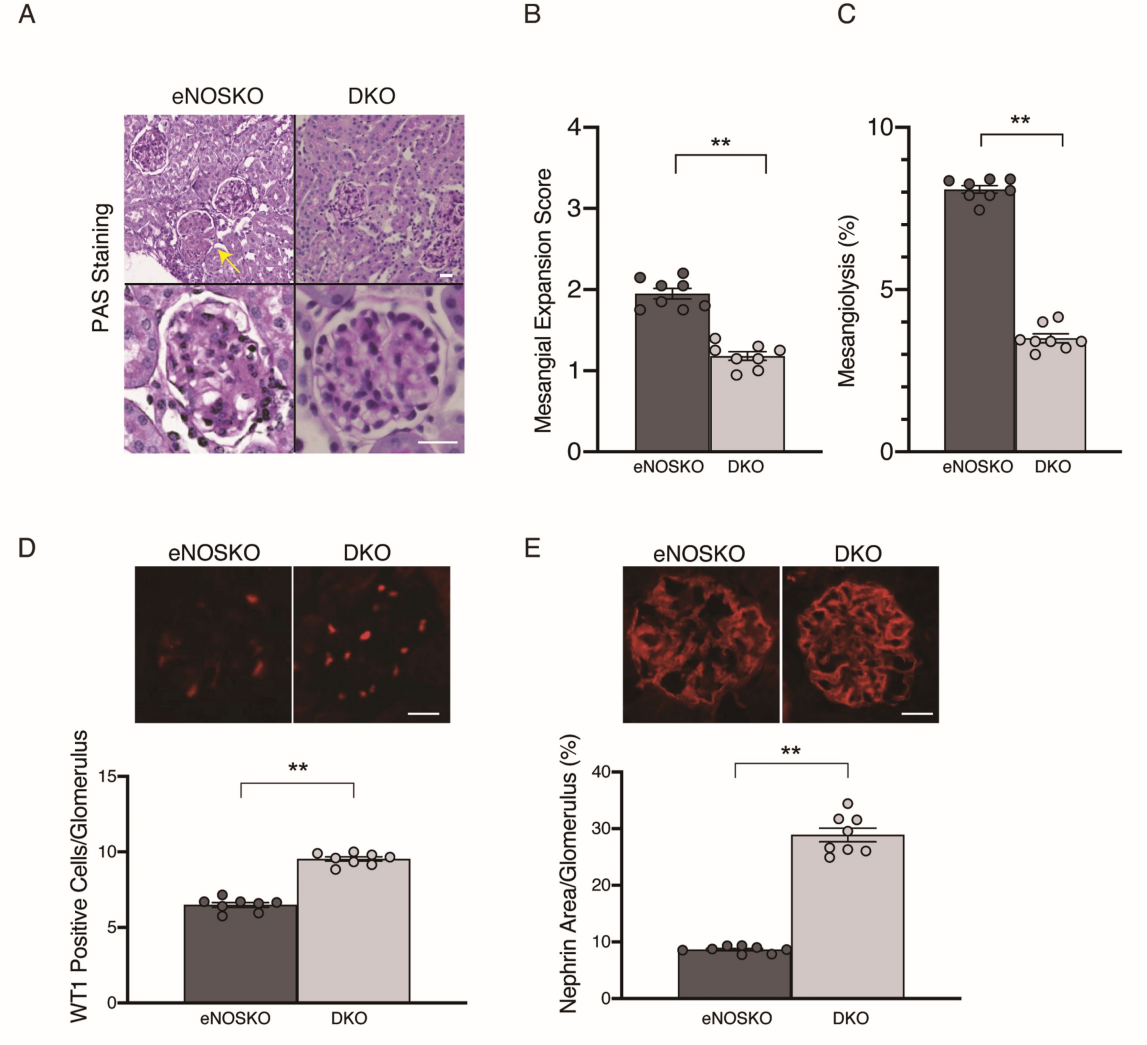
Histological and immunofluorescent evaluation of diabetic PTP1B/eNOS DKO mouse glomeruli A: Glomerular pathology. B: Mesangial expansion score. C: Mesangiolysis (% glomeruli). D: Number of WT1-positive cells (podocytes) in glomerular cross-section. E: Nephrin-positive area in glomerular cross-section. The glomerulus indicated by a yellow arrow (panel A) shows mesangiolysis. Data are expressed as mean values ± SEM. **p < 0.01 vs. diabetic eNOSKO group. N=8 per group. Scale bar=20 µm. eNOSKO, eNOS knockout; DKO, double knockout; PAS, Periodic acid-Schiff; WT1, Wilms’ tumor gene 1 protein

Histological and immunofluorescent analyses of glomeruli revealed significantly less mesangial expansion and mesangiolysis, a higher number of WT1-positive podocytes, and increased nephrin immunoreactivity in diabetic DKO mice (Figures 2A-2E).

**Figure 3:**
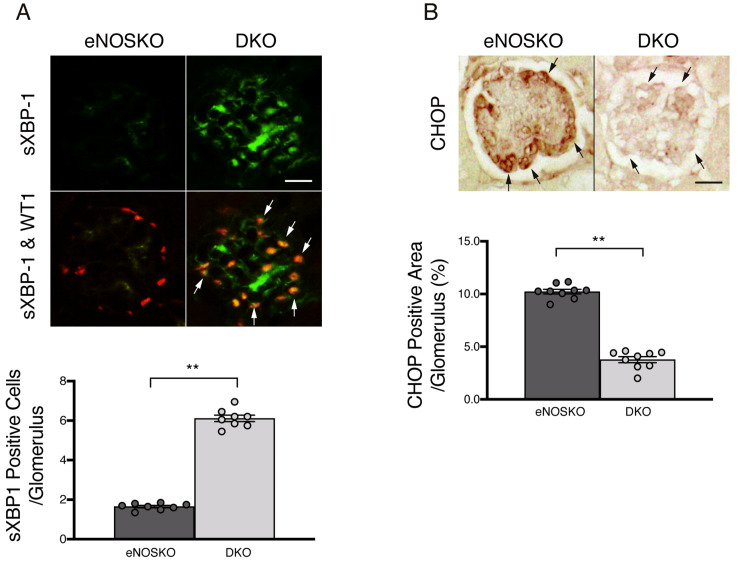
The effects of PTP1B deficiency on ER stress in eNOS-deficient diabetic glomeruli A: Glomerular sXBP-1 immunoreactivity in diabetic PTP1B/eNOS DKO and eNOSKO mice. sXBP-1 was co-immunostained with WT1. B: Glomerular CHOP immunoreactivity. Arrows indicate podocytes (arrows). Data are expressed as mean values ± SEM. **p < 0.01 vs. diabetic eNOSKO group. N=8 per group. Scale bar=20 µm. eNOSKO, eNOS knockout; DKO, double knockout; sXBP-1, spliced X-box binding protein 1; WT1, Wilms’ tumor gene 1 protein; CHOP, C/EBP-homologous protein

Since a recent study showed that insulin signaling increases sXBP-1 and its nuclear localization and reduces CHOP expression in diabetic podocytes, improving their ER stress [14], we further examined sXBP-1 and CHOP expression by immunostaining. As shown in Figures 3A-3B, diabetic DKO mice showed increased expression and nuclear localization of sXBP-1 and reduced CHOP immunoreactivity in glomeruli, especially in podocytes, suggesting enhanced insulin signaling in podocytes.

## Discussion

PTP1B deficiency substantially attenuated glomerular injury in diabetic eNOSKO mice. Recent studies have shown a pivotal role of insulin signaling in podocyte health [15]. Podocyte-specific insulin receptor KO mice develop evident albuminuria and severe podocyte injury, glomerular type IV collagen accumulation, glomerular basement membrane thickening, and glomerulosclerosis, recapitulating DN [16]. Madhusudhan et al. showed that insulin signaling through p85α and p85β subunits of phosphatidylinositol 3-kinase promotes nuclear localization of sXBP-1 and controls ER stress in podocytes [14]. The reduction in insulin signaling in combination with hyperglycemia decreases sXBP-1 and concurrently increases CHOP expression, impairing adaptive sXBP-1 signaling and causing maladaptive ER stress in podocytes. Garner et al. demonstrated that enhancing insulin signaling by PTP1B knockdown protects against ER stress in cultured podocytes, including increased CHOP expression and apoptosis [17]. However, whether PTP1B inhibition attenuates ER stress in diabetic podocytes in vivo remains unknown. Our data suggest that PTP1B deficiency enhances insulin signaling in the conditions of hypoinsulinemic diabetes and eNOS deficiency, increases adaptive sXBP-1 signaling, reduces maladaptive CHOP expression, and attenuates diabetic podocyte injury in vivo. However, it was difficult to prove the enhancement of insulin signals (including phosphorylation of insulin receptor or insulin receptor substrate 1) in podocytes in the STZ model due to the hypoinsulinemic state. It is of note that PTP1B deficiency did not alter blood glucose levels in STZ-eNOSKO mice. Ito et al. also demonstrated that PTP1B deficiency does not change blood glucose and body weight in STZ-induced diabetic mice [18]. PTP1B deficiency may be insufficient for reducing blood glucose in the STZ model due to the low insulin level.

PTP1B deficiency also reduced mesangial expansion and mesangiolysis in diabetic eNOSKO mice. Although the underlying mechanisms are currently unknown, given that PTP1B activates Src kinase that plays an important role in mesangial matrix accumulation [19,20], attenuated mesangial expansion may be due to the reduction of Src activity. In endothelial cells, PTP1B reduces vascular endothelial growth factor receptor-2 (VEGFR2) signaling [21]. It also contributes to endothelial ER stress via protein kinase RNA-like endoplasmic reticulum kinase (PERK) [22]. Therefore, PTP1B deficiency may reduce glomerular endothelial cell damage by increasing VEGFR2 activity or reducing ER stress, leading to a reduction of mesangiolysis.

PTP1B has some functions other than the regulation of insulin signaling. It activates Src kinase [23,24], mediates ER stress response independent of insulin signals [22,25], and regulates mitochondrial biogenesis, dynamics, and function [26,27]. Therefore, PTP1B deficiency may attenuate these pathological events in diabetic podocytes. Further studies are required to elucidate the detailed mechanisms underlying the protective effects of PTP1B inhibition on podocytes. PTP1B is expressed ubiquitously; therefore, it would also be important to assess its effects on other glomerular and renal cells, inflammatory cells, and tubulointerstitial lesions.

There are difficulties in developing highly selective PTP inhibitors. However, alternative strategies have recently been developed, including allosteric inhibition and antisense oligonucleotides [28,29]. Currently, highly selective and potent PTP1B inhibitors are being developed for diabetes treatment. The elucidation of renal effects of PTP1B inhibition would provide new treatment strategies for DN.

## Conclusions

The present study demonstrates that PTP1B deficiency substantially reduces glomerular injury in eNOS-deficient diabetic mice without altering blood glucose and blood pressure levels, including albuminuria, mesangial expansion, mesangiolysis, and reduction of podocyte numbers and nephrin expression. Our data also demonstrates that PTP1B deficiency greatly increases sXBP-1 nuclear localization and reduces CHOP immunoreactivity in eNOS-deficient diabetic glomeruli, especially in podocytes, suggesting enhanced insulin signaling and improved endoplasmic reticulum stress in podocytes as a possible mechanism. These findings demonstrate that PTP1B mediates advanced diabetic glomerular injury induced by eNOS deficiency, shedding light on PTP1B as a therapeutic target for DN.
